# Current and future climatic regions favourable for a globally introduced wild carnivore, the raccoon *Procyon lotor*

**DOI:** 10.1038/s41598-019-45713-y

**Published:** 2019-06-24

**Authors:** Vivien Louppe, Boris Leroy, Anthony Herrel, Géraldine Veron

**Affiliations:** 1 Institut de Systématique, Evolution, Biodiversité (ISYEB), Muséum national d’Histoire naturelle, CNRS, Sorbonne Université, EPHE, Université des Antilles, 57 rue Cuvier, CP 51, 75231 Paris, Cedex 5 France; 2 Unité Biologie des Organismes et Ecosystèmes Aquatiques (BOREA UMR 7208), Muséum National d’Histoire Naturelle, Sorbonne Universités, Université de Caen Normandie, Université des Antilles, CNRS, IRD, Paris, France; 3 Département Adaptations du Vivant (FUNEVOL, UMR 7179), Muséum National d’Histoire Naturelle, CNRS, Paris, France

**Keywords:** Invasive species, Climate-change ecology

## Abstract

Invasive species are considered as one of the major threats to biodiversity and represent a major challenge in the conservation of natural ecosystems, in preventing damage to agricultural production, and human health risks. Environmental Niche Modelling has emerged as a powerful tool to predict the patterns of range expansion of non-native species and to direct effective strategies for managing biological invasions. The raccoon, *Procyon lotor*, is a wild mesocarnivore presenting a high adaptability and showing successful introduced populations worldwide. Here, we modelled the current and future climatically favourable areas for the raccoon using two protocols, based on data sets filtrated in geographic and environmental spaces. Projections from these models show extensive current favourable geographical areas covering extensive regions of temperate biomes. Moreover, predictions for 2050 reveals extensive new favourable areas north of the current favourable regions. However, the results of the two modeling approaches differ in the extent of predicted favourable spaces. Protocols using geographically filtered data present more conservative forecasts, while protocol using environmental filtration presents forecasts across greater areas. Given the biological characteristics and the ecological requirements of a generalist carnivore such as the raccoon, the latter forecasts appears more relevant and should be privileged in the development of conservation plans for ecosystems.

## Introduction

The comprehension of the relationships between organisms and their environment, and an understanding of the dynamics of species distributions is crucial to be able to predict species responses to the current environmental crisis. The recent development of computational and statistical tools allowed the emergence of new techniques to model ecological niches and potential species distributions (e.g. refs^1–7^). By associating geo-referenced occurrence data and environmental variables, Environmental Niche Modelling (hereafter called ENM) (also known as Species Distribution Modelling), makes it possible to evaluate the potential ecological niche of a species, and thus to identify the geographical areas that are favourable to its presence^8–11^. Associated with future climate change scenarios, ENMs also make it possible to predict the evolution of these favourable areas within a temporal framework^12–19^. In this way, ENMs open up a wide range of research, especially with regard to biodiversity conservation issues, notably through the study of rare or threatened species^20–30^, or conversely, of invasive alien species (e.g. refs^31–33^).

Invasive alien species are now considered as one of the major threats to biodiversity and represent a prime challenge in the conservation of natural ecosystems. The present study focuses on a globally introduced mammal, the northern raccoon (*Procyon lotor*). The raccoon is a carnivore native to the North American continent. Yet, this species has been moved to different areas through the pet trade and for commercial purposes (exploitation of its fur). These activities have led to its introduction and spread in many regions worldwide. The species has been present in several Caribbean islands since the 17th century^34^, in Japan since the 1960s^35^, in Azerbaijan and Iran since 1991^36^, but also in several European countries where populations are currently expanding^37^. Since its introduction in the 1930s in Hessen, Germany, the species has quickly dispersed to bordering countries, and other independent introduction events have also been reported. The raccoon was observed in France as early as 1934, in the Netherlands in 1960, in Austria in 1974, in Switzerland in 1976, and in Luxemburg in 1979^38^. Raccoons have also been observed in Denmark, Belgium, Czech Republic, Poland^38^, and very recently in Spain^39^ and Italy^40,41^.

The raccoon appears well adapted to urban environments, which increases the connectivity between occupied natural habitats, and may favour its successful establishment in introduced regions. The expansion of populations may also be favoured by an extremely versatile diet^37,42^, which also weakens the impact of potential competitors. The species may represent a threat, particularly to vulnerable ecosystems such as insular environments. Known to impact marine turtle^43^ and bird populations^44^ on several islands in its native distribution range, the raccoon has often been suspected to impact bird^45^, turtle and iguana populations^46,47^ of several Caribbean islands where the species has been introduced. However, its influence as a competitor or predator in native ecosystems, notably in continental environments, remains poorly documented^37,48^, and the impact of its introductions remains to be rigorously assessed. The raccoon is also considered as an agricultural pest, responsible of extensive damage to crops, orchards, and livestock feed^49,50^. Finally, the species is a recognized vector of diseases such as rabies and nematode-mediated pathologies^51–56^, raising concern for wildlife managers and agricultural producers.

ENMs have quickly emerged as important tools in identifying biotic and abiotic environmental factors that may influence the spread and distribution of non-native species^16,57–62^. The use of ENMs in invasive alien species studies has greatly contributed to their development^63,64^, and also highlighted various constraints and difficulties inherent to this technique^65–67^. While some issues lie in the quality of the data (taxonomic identification errors, sampling bias, geo-referencing precision), major difficulties arise from methodological limitations, such as the selection of the spatial framework of study, model-based variations and uncertainty, or the choice of methods for model evaluation^68–73^. Moreover, most ENM techniques require absence data for model calibration. Yet, these data are often difficult to obtain and are seldomly available. Hence, virtual absences, called pseudo-absences, have been proposed as an alternative. These pseudo-absences are randomly generated over the entire available environmental space^74^. However, this method presents a risk of generating false absences, largely impacting model performance and the identification of favourable areas^75^. Thus, different alternative methods have been explored, generally relying on the application of buffer distances from known presence localities^76–79^. Recently, methods relying on the identification of the environmental space occupied by the species, in order to generate pseudo-absences in the unoccupied environmental space, have demonstrated significant improvement of model performance^80^.

Another methodological difficulty associated with ENMs, particularly in invasive species studies, lies in the choice of data used for the modelling. Early approaches suggested the use of native occurrences in model calibration, and projection of these models into invaded regions^81^. This approach relies on the principle of niche conservatism, which assumes the retention of inherited niche-related ecological traits over time and space^82^. However, this assumption has been highly debated over the past decade, as niche shifts have been reported in invasive plants (e.g. refs^83–85^), insects^86,87^, reptiles^88,89^, amphibians^90^, and birds^91^. However, some authors suggested that these niche shifts might result more from a partial representativeness of the fundamental niche in the native range (for example due to dispersal capacities, competition, predation, or the absence of all favourable climatic conditions in the native area), rather than from true evolutionary niche shifts^92–94^. Alternatively, some authors suggested that niche conservatism could be restricted to short-to-moderate time spans and a lower taxonomic ranks^95^, while niche shifts might occur at evolutionary timescales, with variation among higher taxonomic ranks^96,97^. Another idea is that particular dimensions (e.g. temperature, precipitations, annual and seasonal variations) could be more conserved than others, and that niche conservatism may not encompass the entire niche^98–101^. Although this debate has not yet reached a consensus, considering niche conservatism or not is particularly important regarding the selection of data used in modelling. In fact, if niche conservatism is not considered, modelling favourable spaces of an introduced species requires model calibration using both native and non-native range occurrences^58,102,103^. Moreover, considering that niche shifts are common in invasive species has profound consequences on the interpretation of potential distributions predicted by ENMs^94,104,105^. Consequently, evaluation of niche conservatism represents a crucial step in invasive species niche modelling^103,106^.

In this study, our first objective is to compare the bioclimatic space occupied by the raccoon in its native range versus the three non-native areas where it has substantially spread (the Caribbean region, Europe, and Japan). Our second objective is to model the current and future climatically favourable areas on the basis of environmental niche models. To this end, we applied two modelling protocols, a first one based on a geographical filtration of species presences and a random selection of pseudo-absences, and a second one based on an environmental filtration of species presence and selection of pseudo-absences outside the environmental space occupied by the species. We applied an ensemble modelling procedure based on nine statistical models, two future scenarios based on the extreme ends of the Representative greenhouse gas Concentration Pathways (RCP) scenarios, and a consensus of three Global Circulation Models (GCM).

## Results

### Environmental space comparisons

**Figure 1:**
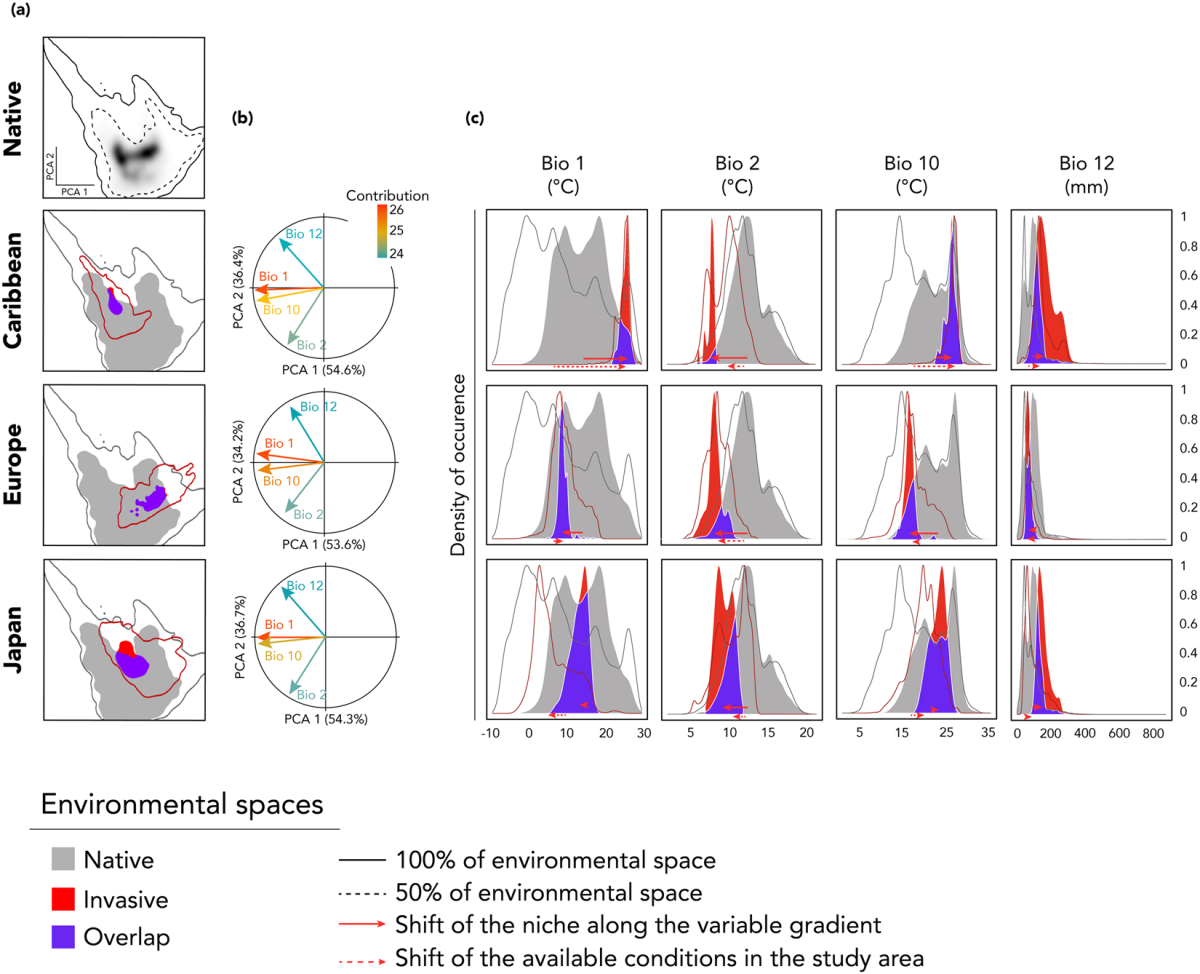
Analyses of environmental space shifts between the native range and the three main regions of introduction of *Procyon lotor*. (**a**) Environmental occupancy of *P. lotor* in native and non-native ranges along the two first axes of the PCA. Grey to black gradients in native range graph represent the density of occurrence. Solid lines represent 100% of the available environmental space. Dashed lines represent 50% of the environmental space. (**b**) Contribution of the bioclimatic variables to the two first axes of the PCA, and percentage of inertia explained by the two axes. (**c**) Occupancy of the niche along each variable gradient. A solid red arrow represents the shift of the non-native niche along the variable gradient. A dashed red arrow represents the shift of the available conditions in the non-native range. Solid lines represent 100% of available environmental space.

Our results indicated very little overlap between the native niche and either of the Caribbean, the European, and the Japanese niches (Caribbean: D = 0.01; European: D = 0.08; Japanese: D = 0.16; Table 1; Fig. 1). Moreover, niche similarity was rejected as D indices fell outside the 95% confidence interval of simulated values (Table 1). However, niche equivalence was rejected only for the Caribbean niche. In addition, we found that in all invaded areas, niches presented low expansion, but high unfilling compared to native niches, indicating that the species has not colonized all the possible environmental conditions shared with the native niche or that those conditions were absent from the non-native range (Table 1; Fig. 1).

**Table 1:** Tests of niche overlap, niche equivalency and niche similarity between the native niche and the niches of the three main non-native regions of *Procyon lotor*.

	Overlap D	Equivalency *P*_*D*_	Similarity Inv. to Nat. *P*_*D*_	Expansion	Unfilling
Caribbean	0.01	1	0.66	0.06	0.96
Europe	0.08	<0.01	0.32	0	0.65
Japan	0.16	<0.01	0.18	0.17	0.74

### Bioclimatic niche model

**Figure 2:**
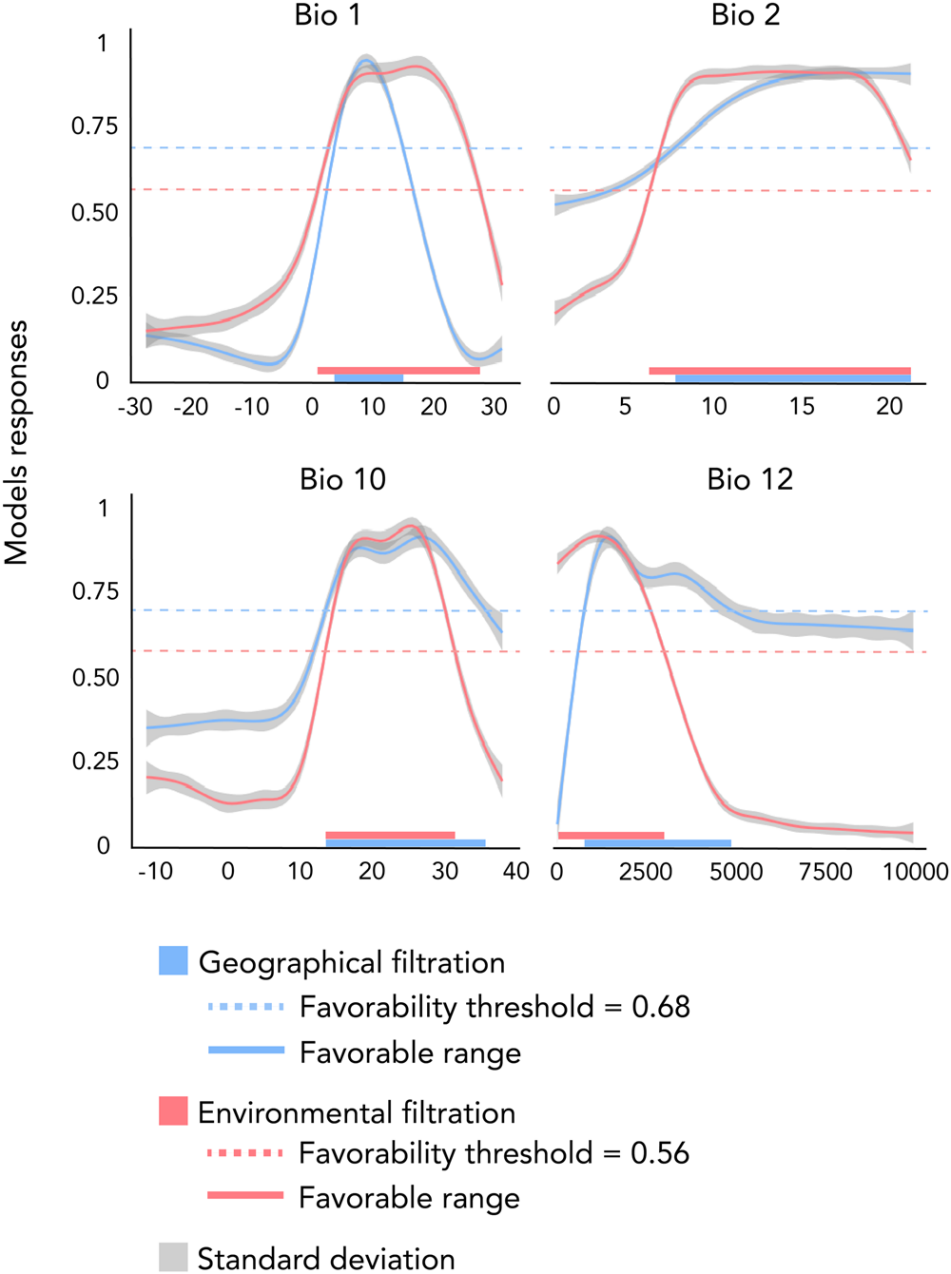
Response curves of the favourability value predicted by the models used in the models with Geographic Filtration (blue) and Environmental Filtration (red) approaches. Dashed blue and red lines represent favourability threshold identified for predictions of respectively GF and EF approaches. Solid blue and red lines represent the favourable range along the variable gradient.

Modelled niches differed between the EF and GF approach, with sharper responses modelled for all variables except bio1 under the EF approach (Fig. 2). Most importantly, the EF approach identified a clear response to bio2 and bio12, whereas the GF approach identified very shallow responses. Conversely, EF niches showed a broader tolerance to bio1 (a plateau between 8 and 20°C) than GF niches (a single optimum at 8 °C).

**Figure 3:**
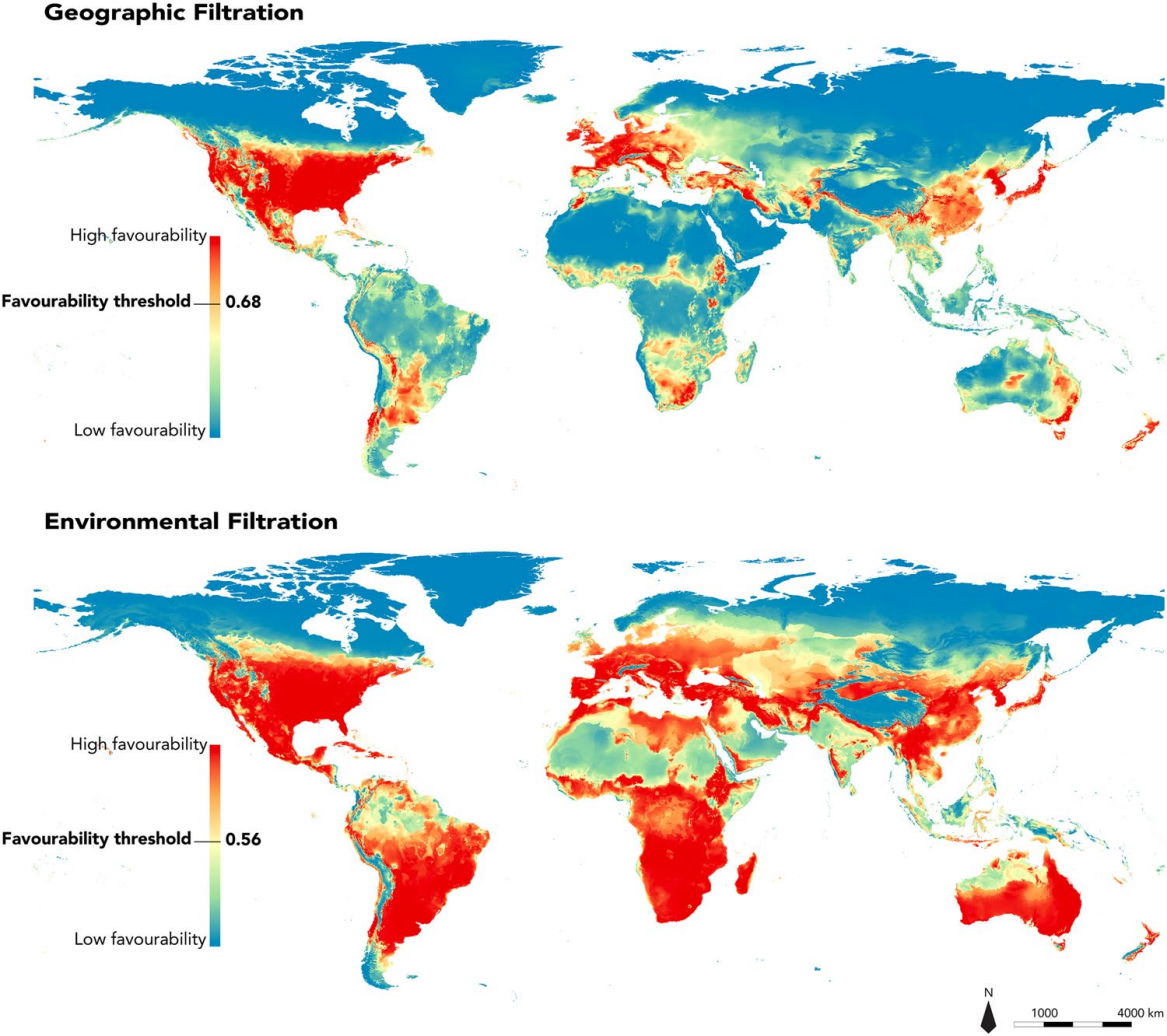
Projection of global bioclimatic favourability for *Procyon lotor*, predicted through Geographical Filtration and Environmental Filtrations approaches.

**Figure 4:**
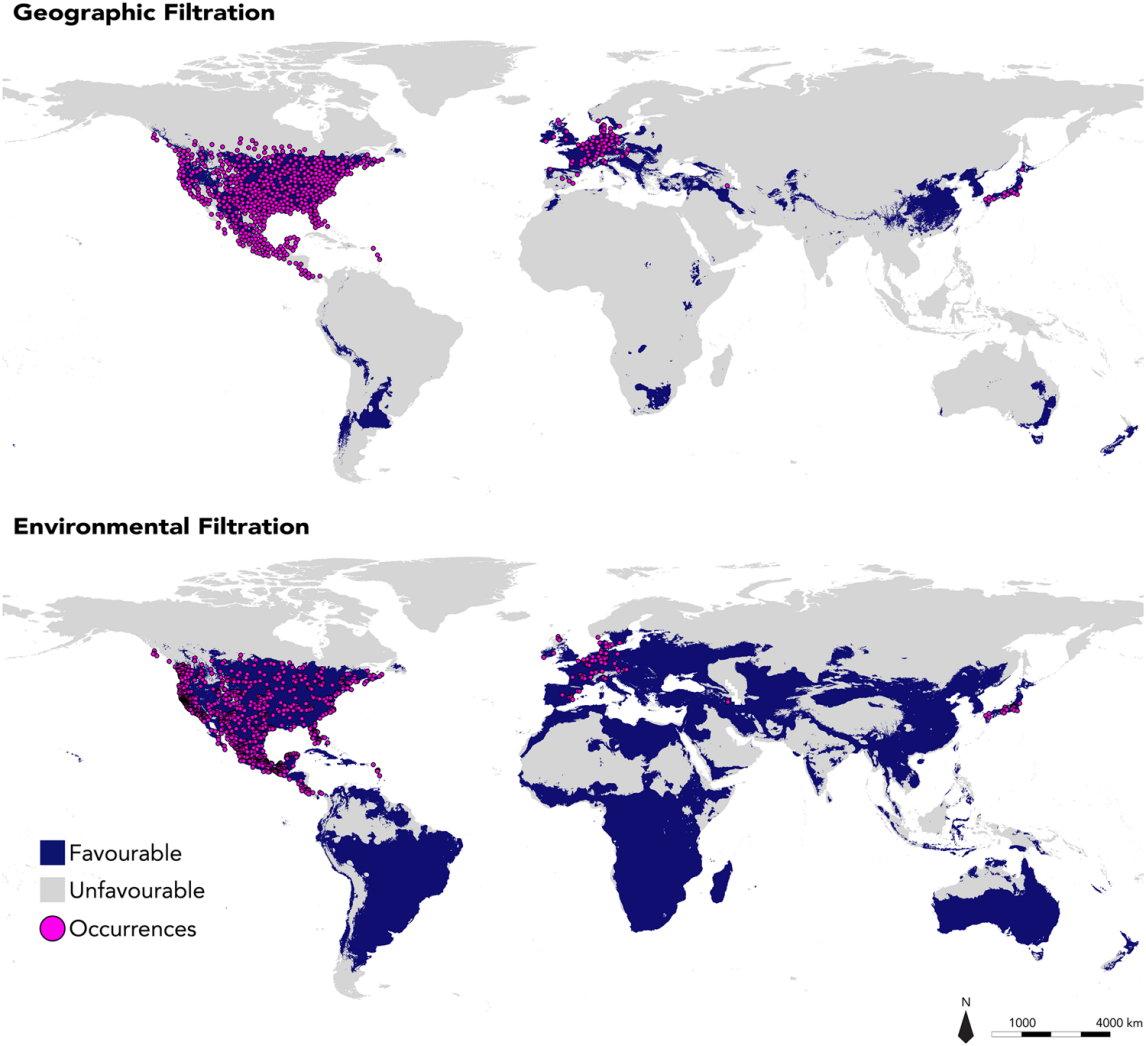
Projection of global bioclimatic favourable areas for *Procyon lotor*, predicted through Geographical Filtration and Environmental Filtrations approaches. Pink circles represent occurrences used in the modelling.

As expected, both EF and GF approaches predicted similar favourable areas for the species over most of the temperate and sub-tropical regions (Figs 3, 4 and S1). However, the EF approach also predicted substantially larger favourable areas than GF, representing respectively 44.2% and 11.9% of all terrestrial areas (Figs 3, 4 and S1). Interestingly, the EF approach predicted that most tropical areas in Africa, and South-East Asia were favourable for the raccoon, whereas GF results were limited to temperate and dry areas of the world.

**Figure 5:**
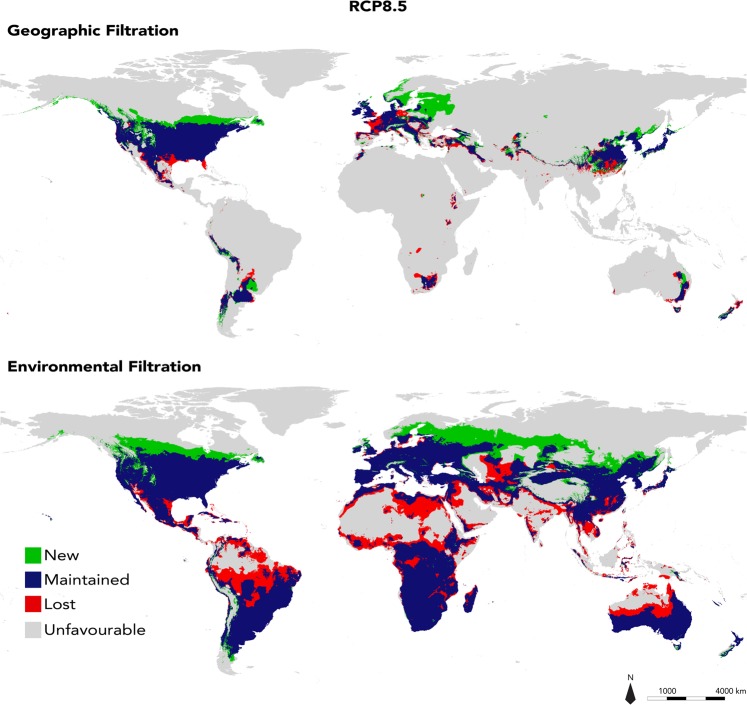
Predicted favourable range change for *Procyon lotor* by 2050 according to scenario RCP8.5. Unfavourable: areas that are currently unfavourable remain unfavourable in the future; Lost: areas currently favourable that will lose their favourable nature in the future; Maintained: areas that are currently favourable and will still be favourable in the future; New: areas that are currently not favourable but would become favourable in the future.

Both approaches present important differences in favourable areas at 2050. Favourable spaces are predicted to respectively increase by 18.5% (with RCP2.6; Supplementary Fig. S2) or 17.6% (with RCP8.5; Fig. 5) of its actual size with the GF approach, with broad new areas increasing from the northern border of the native range and in north-east Europe. Alternatively, the EF approach predicted a slight contraction of favourable areas by 2050, varying between 2.1% (RCP2.6; Supplementary Fig. S2) to 5.5% (RCP8.5; Fig. 5) of actual favourable areas. Lost spaces are distributed along tropical and sub-tropical regions, resulting in a shift of favourable spaces toward the northern regions of North America and Eurasia. Finally, both approaches predict that currently occupied areas are preserved in 2050.

## Discussion

This study presents the most complete analysis of the bioclimatic envelope favourable to the presence of the globally introduced carnivore *Procyon lotor*, and the first forecast of areas favourable to the species at a global scale using multiple modelling techniques and methods. Our results highlight the variability in environmental conditions between the native range and the regions where the raccoon was introduced. Consequently, niche models were computed with occurrences sampled from both native and non-native areas. These models created using two distinct methods demonstrate the high variability in results arising from presence selection and pseudo-absence generation techniques. Accordingly, projections for 2050 reveal major differences in favourable space from one model to another. However, both approaches highlight the ecological plasticity of the raccoon, showing large spaces favourable to the species at a global scale. Furthermore, projections for 2050 predict a high stability of current favourable areas, particularly in regions where the raccoon is already present.

Niche conservatism tests performed for the three main regions colonized by the raccoon showed contrasting results regarding niche equivalence and niche similarity. Despite the fact that equivalency tests suggest that the European and the Japanese niches are relatively conserved, similarity tests reveals significant differences between the three regions of introduction and the native niche. Yet, a common factor appears to explain the differences between the native niche and the three colonized areas. As shown in several other invasive species^107,108^, these differences are explained more by unfilling than by expansion. Unfilling may be the result of different processes including ongoing colonization, slow dispersal, or the impossibility to reach new areas^98,105,109,110^. Unfilling values for the Caribbean and Japan niches may then result from the peculiar dispersion and distribution constraints involved by insular environments. On the other hand, unfilling values for the European region can be explained, at least in part, by the ongoing expansion of the populations.

The differences in environmental conditions between the different areas where the species is present, particularly in the Caribbean region, show the necessity of taking into account occurrence data from both native and non-native areas in order to model the bioclimatic niche of the raccoon as accurately as possible. Moreover, considering that the niche of a species is conserved from a region to another implies, in absolute terms, that the niches are at equilibrium, i.e. that the species is present in all the available niches in the introduced area, and absent in all regions not in accordance with its ecological requirements. However, regarding the raccoon, as the European populations are still expanding, the possibility of niche expansion in later stages of colonization cannot be excluded^111^. In that respect, the niche of the raccoon cannot be considered conserved in the different regions of introduction.

In this study, two modelling approaches were used. These two approaches differ by the selection of occurrence data and by the method of generation of pseudo-absences. The first approach, GF (Geographical Filtration), relies on using occurrence data that are spatially thinned and pseudo-absences that are randomly generated within all the available environmental space. The second approach, EF (Environmental Filtration), is based on an environmental filtration of occurrence data and the use of pseudo-absences randomly generated outside of the environmental space occupied by the species.

Consequently, model responses differ substantially. Overall, response curves obtained through the EF approach display a better normalization and a reduced variance. This results in projections diverging particularly with regard to the extent of regions identified as favourable. As expected, the GF approach generates smaller favourable areas than the EF approach. This difference may arise from the fact that model responses for bio1 present a substantially wider range of favourable temperatures with the EF than with the GF approach. In addition, the geographical filtration, particularly subject to sampling biases, maintains an artificial heterogeneity in the representation of the favourable environmental conditions that may also explain the differences between the predictions of the two approaches. Finally, the random generation of pseudo-absences within the entire available geographical space inevitably leads to the generation of pseudo-absences in sites presenting known favourable environmental conditions. For example, pseudo-absences can be selected within the current range of the species. This results in a confounding influence on the identification of favourable conditions by the models, which might be more important in the case of species presenting a tolerance to a broad range of bioclimatic conditions such as the raccoon. The EF approach, by selecting pseudo-absences within conditions where the species has never been reported, eliminates this bias and allows for a better characterization of the species niche.

Results obtained through the EF approach also appear to be better matched to the ecology of the species. The raccoon is present in a wide range of habitats, elevations and climatic conditions. Raccoon populations thrives in regions as diverse as the Caribbean mangroves, the European temperate forests or the Laurentian mixed forests of North America. Thus, the restricted temperature optimum as well as the limited favourable areas identified through the GF approach appear inconsistent regarding the ecological requirements of an ubiquitous mammal such as the raccoon. Moreover, the binary projections, resulting from the GF approach, and highlighting the highly favourable space, present a very incomplete coverage of the regions where the presence of the raccoon is proven, particularly in its native distribution range. Through the example of the raccoon, it appears that an approach based on a geographical filtration of the presence/pseudo-absence data can lead to an underestimation of the area favourable for the studied species; which may have important consequences in a context of biodiversity management, and particularly in regions of introduction of alien species.

Our results reveal the raccoon’s tolerance to a very wide range of bioclimatic conditions, resulting in vast areas favourable to the species at a global scale. By highlighting the regions that are most likely to be colonized, these projections represent important tools in orienting and optimizing the monitoring efforts of the species. Since the first introductions in Europe in the early 20th century, raccoon populations have thrived and are currently present in all Western European countries. Some populations are also rapidly expanding from the center of Europe to the east, where our results identify highly favourable areas, extending in to Russia and the Middle East. Similarly, our results indicate highly favourable areas covering a large number of islands, such as different islands of the Caribbean and the Japanese archipelago, but also Madagascar, New Zealand, or Tasmania. Island environments are particularly vulnerable to the colonization of invasive alien species, notably carnivores, which are often poorly represented in native ecosystems^112^. The impact of introduced raccoons may thus be more damaging on islands where native fauna has been little exposed to predation, and where raccoons themselves are exposed to little predation and competition.

Invasive species management is a priority for the conservation of ecosystems and biodiversity. ENMs represent particularly effective tools to grasp the distribution of these species at different spatial and temporal scales as they can take into account the ongoing and future environmental change. Our analyses of favourable geographic space to the raccoon at the horizon 2050 predict, with both approaches, a global stability of current favourable areas (Fig. 5), with the exception of the Caribbean islands where a potential extirpation of the species may take place by 2050. Moreover, new favourable areas appear to extend broadly in northern regions in continuity with maintained favourable spaces. These results were expected given the fact that climate change scenarios predict a global warming of arctic regions, and that temperature was identified as the most influential bioclimatic factor for the raccoon (three out of the four environmental variables used in modelling correspond to temperature measures; Supplementary Fig. S3). However, while lost space is limited and fragmented in GF approach projections, it appears more pronounced with the EF approach. Thus, projections with the GF approach predict a clear expansion of favourable areas, whereas projections with the EF approach show a slight contraction and a northward shift of favourable spaces. Lost spaces are principally located at tropical latitudes, and newly favourable areas mainly cover regions to the north of current favourable areas. These observations are similar for both representative greenhouse gas concentration pathways scenarios (RCP2.6 and RCP8.5), but more pronounced in the case of RCP8.5. In accordance with the EF approach predictions, a northern shift has also been predicted for the species in its native range, as is the case for other North-American carnivores^113^ and northern European species^114,115^. In northern Europe, the development of spaces that are favourable to the raccoon may represent an additional threat to boreal ecosystems, where temperature changes are predicted to be the highest^116^. Arctic species are exposed to fewer competitors, predators, parasites and diseases^117^. Potentially affected by climate change, these species can be even more vulnerable to the introduction of new competitors, predators and potential vectors of disease such as the raccoon. This risk appears all the more likely as carnivores might be better able to keep pace with climatic changes than other mammals^118^.

In conclusion, the raccoon presents a tolerance to a very wide range of bioclimatic conditions resulting in extensive regions currently favourable to the species. Moreover, predictions for 2050 reveals wide newly favourable areas north of the current favourable regions. However, differences in the extent of predictions between the two modelling approaches used in this study reveals the importance of the selection of presence and pseudo-absence data. This study demonstrates the importance of data selection and processing in ENM modelling given their crucial role in our understanding of species distribution dynamics and in the development of conservation plans for ecosystems.

## Methods

### Species occurrence data

Occurrences of *P. lotor* were compiled from the online databases VertNet and GBIF, in addition to the databases of the Guadeloupe National Park, Martinique Regional Park, Saint-Martin Natural Reserve, French national wildlife organizations, recent literature, and personal observations. Only recent observations (from 1950 to the present day) with complete and precise locations were selected, giving a total of 20425 occurrences (Supplementary Table S1). All occurrence records were aggregated into 0.08° cells corresponding to the resolution of environmental variables, resulting in a total number of 5922 records (Supplementary Fig. S4).

### Environmental data

Current and future favourable environmental envelopes for *P. lotor* were calculated using 19 bioclimatic variables (derived from temperature and precipitation measures; Table 2), averaged for the period 1950–2000 from the WorldClim 1.4 database^119^. A 5 arc-minute spatial resolution (approximately 9 kilometers at the equator) was selected for all bioclimatic variables.

**Table 2:** Codes and descriptions of the bioclimatic variables and General Circulation Models selected for use in our models.

	Code	Description
Bioclimatic variables	Bio 1	Annual mean temperature
	Bio 2	Mean diurnal range (mean of monthly (max temp - min temp))
	Bio 10	Mean temperature of warmest quarter
	Bio 12	Annual precipitation
General Circulation Models	CNRM-CM5	Centre National de Recherches Météorologiques, France
	GISS-E2-R	NASA Godard Institute for Space Studies, U.S.A.
	MIROC-ESM-CHEM	Japan Agency for Marine-Earth Science and Technology; Atmosphere and Ocean Research Institute (The University of Tokyo); National Institute for Environmental Studies, Japan

Future favourable envelopes for *P. lotor* were modeled using climate projections from global climate models (GCM) based on the Coupled Model Inter comparison Project Phase 5 (CMIP5) averaged for the period 2041–2060. Three GCMs were used (CNRM-CM5; GISS-E2-R; MIROC-ESM-CHEM; Table 2), for two Representative greenhouse gas concentration pathways scenarios (RCP): the most optimistic RCP2.6 (with a radiative forcing of +2.6 W/m² for the period 2000–2100), and the most pessimistic RCP8.5 (with a radiative forcing of +8.5 W/m²).

Collinearity and the influence of the 19 bioclimatic variables were tested, using a protocol adapted from Leroy *et al*.^25^ and Bellard *et al*.^61^ (Supplementary Method S1). This allowed for the identification of four non-collinear variables that significantly influenced the distribution of *P. lotor* (Table 2; Supplementary Figs S3 and S5).

### Analysis of niche conservatism

Following the methodology proposed by Warren *et al*.^3^, and further developed by Broennimann *et al*.^103^, we calculated niche overlap, equivalence, and similarity between the native range (4956 occurrences) and the three major areas colonized by *P. lotor*: the Caribbean (39 occurrences), Europe (868 occurrences), and Japan (56 occurrences). Niche overlap was calculated using a PCA approach and calibrated with the four selected environmental variables for each area of interest. PCA scores of the species occurrences on the first two axes were projected onto a grid of cells delineated by the minimum and maximum PCA scores of the environmental variables. Next, a kernel density function was applied to estimate a density of occurrence for each cell of the grid (see Di Cola *et al*.^120^ for methodological details). Thereafter, niche overlap was estimated using Schoener’s D^121^. This index varies between 0 and 1; 0 meaning no overlap, while 1 meaning identical niches. This index was then used to assess niche equivalence and similarity. Occurrences in each compared range were pooled and randomly split into two datasets with equal size as the original dataset. Niche overlap was then estimated with the D index. This procedure was repeated 100 times to create a null distribution. The observed D was then compared to these simulated values, and the null hypothesis of equivalence between niches was rejected when observed values fell outside of the 95% confidence interval of the simulated distribution. Alternatively, niche similarity test investigates whether the niches in the native or invaded range predict one another better than expected by chance. Occurrences in one area were randomly reallocated within their respective available environmental space and simulated niches were compared to the niche of the other area with both indices. This procedure was repeated 100 times to create a null distribution. The observed D was then compared with the simulated values, and the null hypothesis of similarity of the tested niche to the other was rejected when observed values fell outside of the 95% confidence interval of the simulated distribution.

Along with niche overlap test, niche expansion and unfilling were also calculated. The expansion index corresponds to environmental conditions in the invaded area that are absent in the native area. Conversely, unfilling refers to environmental conditions in the native area that are absent in the invaded area. Both indices vary between 0 and 1.

All niche conservatism tests were performed using the package ecospat v2.1.1^122^ implemented in the R software^123^

### Data preparation and pseudo-absence selection

The preparation of presence-only data for ENMs included two important steps. The first one consisted of filtering presence data to reduce autocorrelation and sampling bias (e.g., refs^124,125^). The second one consisted of selecting pseudo-absence data to calibrate models. Here, we applied two different methods to filter presence data and select pseudoabsences: the first method focused on the geographical space (hereafter Geographical Filtration, GF) whereas the second one focused on the environmental space (hereafter Environmental Filtration, EF).

Geographical filtration (GF) is commonly applied in the ENM literature. We used the R package SPTHIN v.2.1–2^125^ to remove duplicated occurrences in a radius of 100 km, and therefore reduced auto-correlation biases. Consequently, 682 occurrences were retained. Next, we randomly sampled an identical number of pseudo-absences across the available geographical space, with three repetitions. This approach does not filter out repeated occurrences in similar climatic conditions, and samples pseudo-absences in areas that can fall within areas favourable to the species. Therefore, we expect this method to produce a modelled distribution that fits more closely the initial distribution of species occurrences, a “lower-bound” estimate of the species distribution as opposed to the Environmental Filtration method described below.

Environmental filtration (EF) focuses on the distribution of data in the space of environmental variables. Varela *et al*.^124^ showed that removing duplicated records in the environmental space consistently improved the quality of model predictions, contrary to geographical filtering. Therefore, we created a gridded environmental space on the basis of the four selected environmental variables in which we projected all the conditions existing in the geographical space. Next, we projected within this environmental space all the presence points of the species and removed all duplicate points per cell, which resulted in a total of 1036 occurrences. To select pseudo-absences within the environmental space, we applied a procedure to avoid selecting pseudo-absences within environmental conditions that are favourable for the species. To do so, we calculated the restricted n-dimensional convex hull of presences, defined as the smallest convex hull encompassing all occurrence points. This restricted convex hull is considered as a proxy of the favourable environmental conditions outside which we randomly generated 1036 pseudo-absences (i.e., in equal number to the filtered occurrences) with three repetitions. Such a procedure is supported by the statistical theory of model-based designs, also known as “D-designs” which are assumed to minimize prediction variance (see Hengl *et al*.^76^). Overall, the environmental filtering of presences will result in a decreased autocorrelation^124^ and the convex hull will minimize the risk to sample pseudo-absences inside favourable conditions. Therefore, we expect this procedure to produce a larger estimate of the species niche than the GF procedure, hence a larger potential distribution. In addition, we consider this approach to produce a better description potential distribution of the species compared to the GF approach as it corrects for biases linked to heterogeneity in sampling intensity and reduces the risks of generating pseudoabsences falling within favourable areas.

### Ensemble modelling process

Model calibration. Nine different modelling techniques were calibrated and evaluated^126–129^: Artificial Neural Networks – ANN^130^ (Ripley, 1996); Classification Tree Analysis - CTA^131^, Flexible Discriminant Analyses – FDA^132^; Generalised Additive Models – GAM^133^; Generalised Boosted Models – GBM^134^; Generalised Linear Models – GLM^135^; Multivariate Adaptative Regression Splines – MARS^136^; MAXimum ENThropy – MAXENT^1^; Random Forests – RF^137^. For each set of presence/pseudo-absence, model calibration was realized with 70% of all data. The remaining 30% were used for model evaluations. Models were calibrated and evaluated three times per set of presence/pseudo-absence. Model calibrations were realized with the R package BIOMOD 2 v3.3–7^4^.

Because our procedure is a presence/pseudo-absence procedure, we did not calculate discrimination capacity metrics (e.g. the area under the receiver operating characteristic curve or the true skill statistics) because (1) these metrics are designed to be calculated on real absences, and (2) they are dependent on prevalence, which can lead to spurious evaluations of ENMs^138,139^. Rather, we evaluated our models with the Boyce index, specifically developed for such data^109,140,141^. The Boyce index assess how much model predictions match the observed distribution of species occurrences. Values of the Boyce index vary between −1 and +1. Positive values indicate a model with predictions that are consistent with the distribution of occurrences in the evaluation dataset whereas negative values indicate a model with predictions that are not consistent with the distribution of occurrences. Boyce index values close to zero mean that the model is not different from a random model. Models with a mean Boyce index higher than 0.7 were selected (Supplementary Fig. S6). Consequently, all models were kept for EF modelling, but MAXENT was removed for GF modelling. These analyses were performed using the R package ecospat v2.1.1^122^.

Forecasts of current and future favourable areas were obtained by ensemble forecasting method. Current and future ensemble forecasts represent consensual projections of the six modelling techniques, obtained through averaged distributions of favourability scores^4,142,143^. To discriminate current favourable and non-favourable areas, and visualize shifts by 2050, probability distributions were transformed into binary projections. These binary projections were obtained using a probability threshold that maximized the Sørensen value^139^. Sørensen indices were calculated using 70% of occurrences as training data, and the remaining 30% as testing data.

## Supplementary information

Supplementary information

Table S1

## Data Availability

Occurrence data are available in Supplementary Table S1 of Supplementary Informations. All climate GIS layers are available as raster grids from the Worldclim database: www.worldclim.org.
