# Supplementary material for: Current and future climatic regions favourable for a globally introduced wild carnivore, the raccoon Procyon lotor
Source: Sci Rep. 2019 Jun 24;9:9174. doi: 10.1038/s41598-019-45713-y (PMC6591328; doi:10.1038/s41598-019-45713-y)
Supplement: Supplementary file 1 — Supplementary information [file 41598_2019_45713_MOESM1_ESM.pdf]

**Current and future climatic regions favourable for a globally introduced wild carnivore, the raccoon *Procyon lotor***

Louppe, Vivien[1]; Leroy, Boris[2] ; Herrel, Anthony[3]; Veron, Géraldine[1]

1. Institut de Systématique, Evolution, Biodiversité (ISYEB), Muséum national d'Histoire naturelle, CNRS, Sorbonne Université, EPHE, Université des Antilles, 57 rue Cuvier, CP 51, 75231 Paris Cedex 5, France.

2. Unité Biologie des Organismes et Ecosystèmes Aquatiques (BOREA UMR 7208), Muséum National d'Histoire Naturelle, Sorbonne Universités, Université de Caen Normandie, Université des Antilles, CNRS, IRD, Paris, France.

3. Département Adaptations du Vivant (FUNEVOL, UMR 7179), Muséum National d'Histoire Naturelle, CNRS, Paris, France.

Supplementary Informations

Table S1

Occurrence data and sources.

*See attached document*

References

Biedrzycka, A., Zalewski, A., Bartoszewicz, M., Okarma, H. & Jedrzejewska, E. The genetic structure of raccoon introduced in Central Europe reflects multiple invasion pathways. *Biol. Invasions* **16,** 1611–1625 (2014).

Duscher, T., Zeveloff, S. I., Michler, F. U. & Nopp-Mayr, U. Environmental drivers of raccoon (*Procyon lotor* L.) occurrences in Austria - established versus newly invaded regions. *Arch. Biol. Sci.* **70,** 41–53 (2018).

Farashi, A., Kaboli, M. & Karami, M. Predicting range expansion of invasive raccoons in northern Iran using ENFA model at two different scales. *Ecol. Inform.* **15,** 96–102 (2013).

Fischer, M. L. *et al.* Multiple founder effects are followed by range expansion and admixture during the invasion process of the raccoon (*Procyon lotor*) in Europe. *Divers. Distrib.* **23,** 409–420 (2017).

Matějů, J. *et al.* Current distribution of *Procyon lotor* in north-western Bohemia, Czech Republic (Carnivora: Procyonidae) K současnému výskytu mývala severního (*Procyon lotor*) v severozápadních Čechách (Carnivora: Procyonidae). **43,** 133–140 (2012).

Mori, E. *et al.* The masked invader strikes again: The conquest of Italy by the Northern raccoon. *Hystrix* **26,** 1–5 (2015).

Figure S1

Projection of standard deviation values of global bioclimatic favourability for *Procyon lotor*, predicted through Geographical Filtration and Environmental Filtrations approaches.

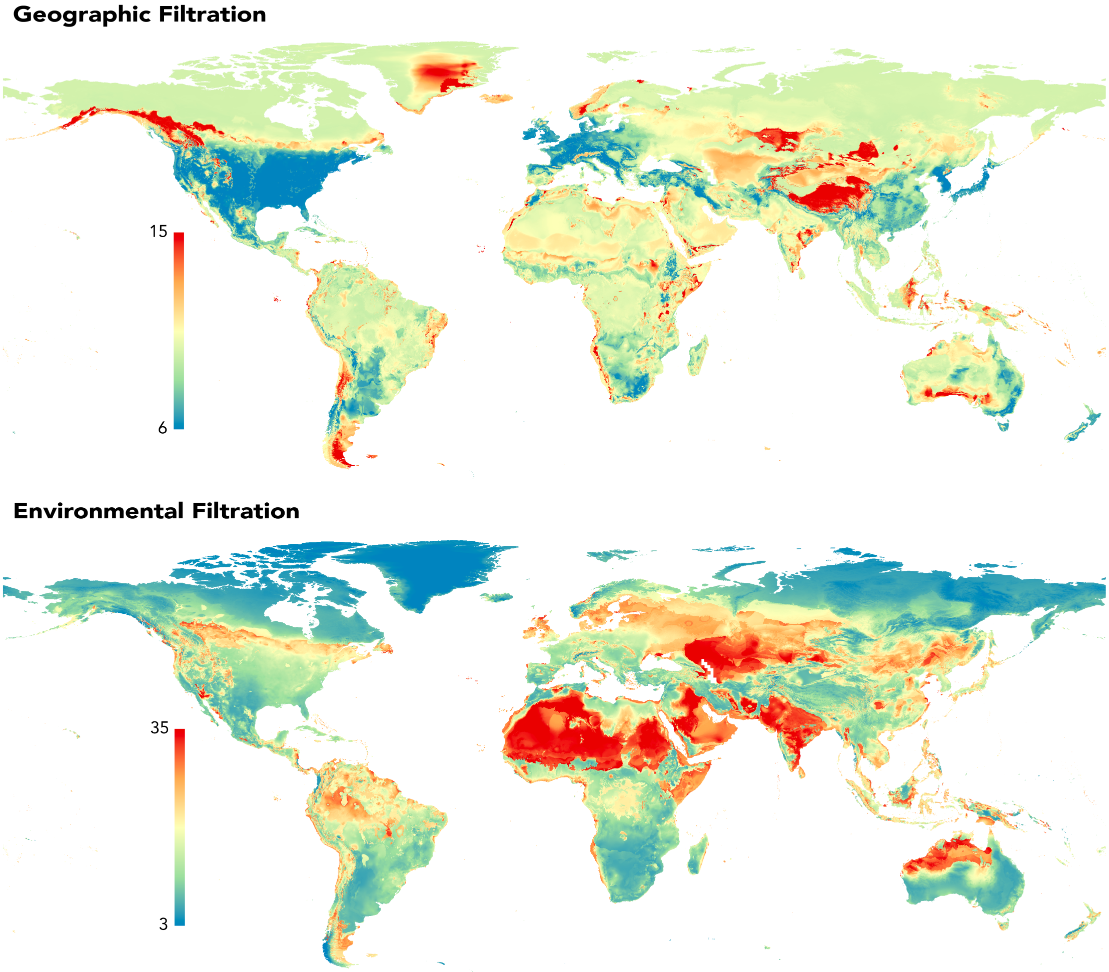

Figure S2

Predicted favourable range change for *Procyon lotor* by 2050 according to scenario RCP2.6. Unfavourable: areas that are currently unfavourable remain unfavourable in the future; Lost: areas currently favourable that will lose their favourable nature in the future; Maintained: areas that are currently favourable and will still be favourable in the future; New: areas that are currently not favourable but would become favourable in the future.

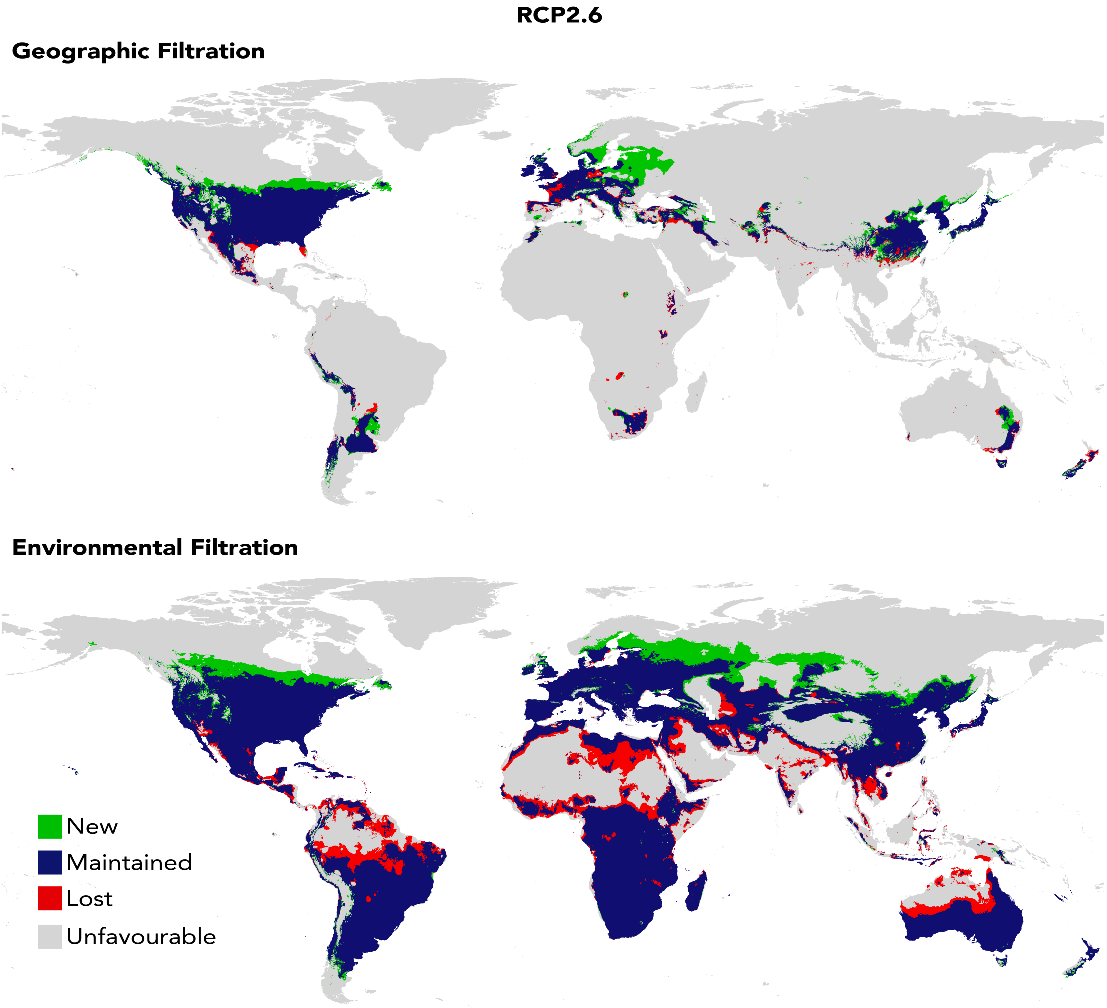

Figure S3

Importance of the environmental variables used in modelling.

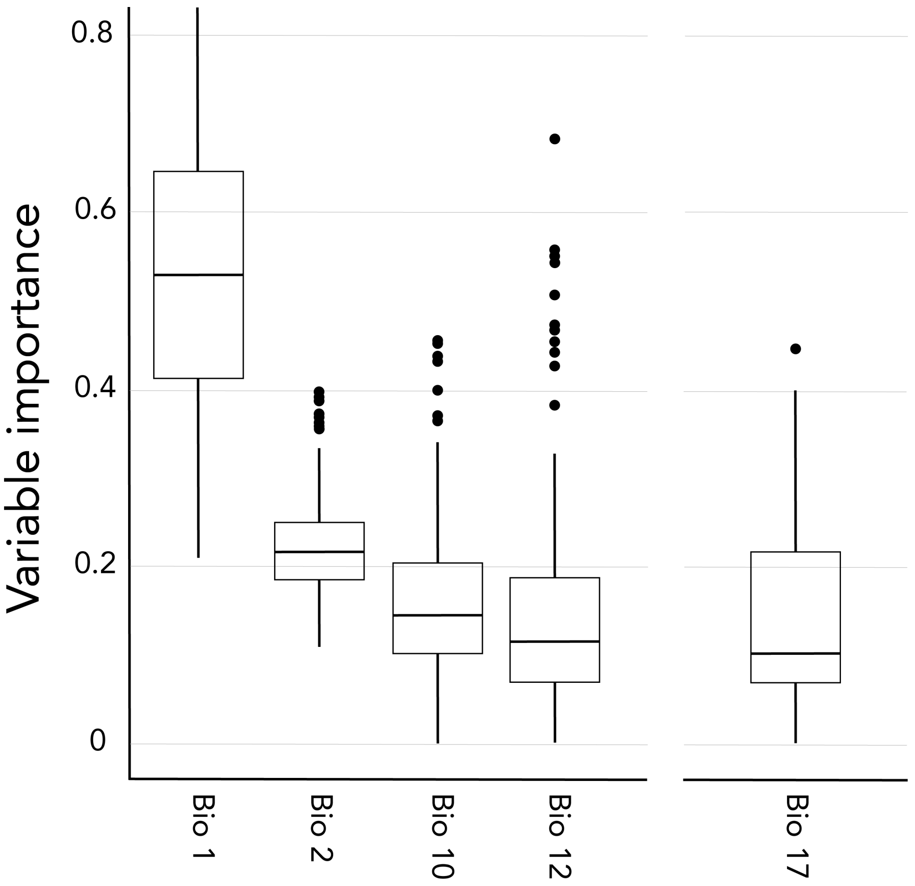

Figure S4

Occurrences aggregated to the resolution of environmental variables.

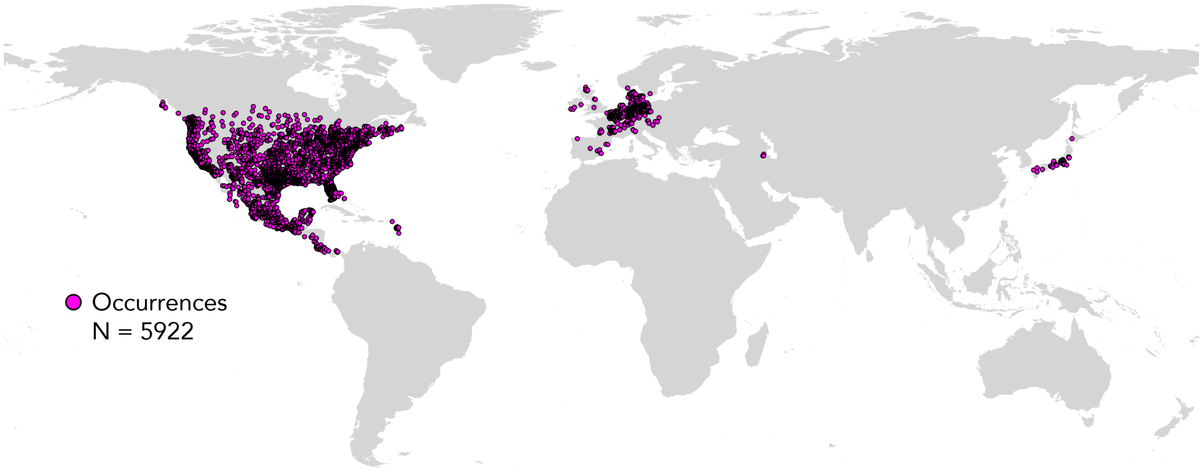

Figure S5

Groups of intercorrelated environmental variables.

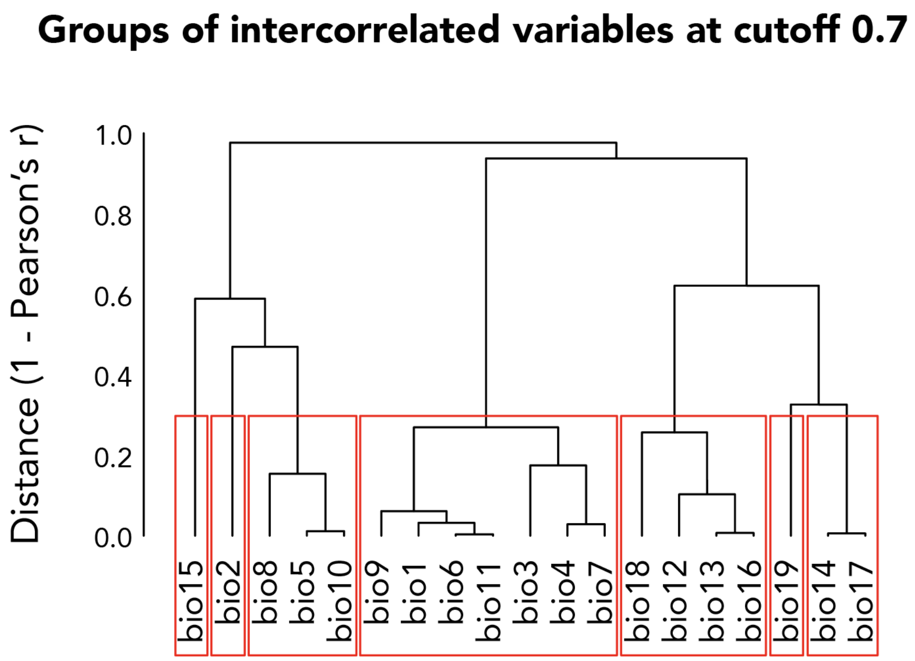

Figure S6

Boyce index values.

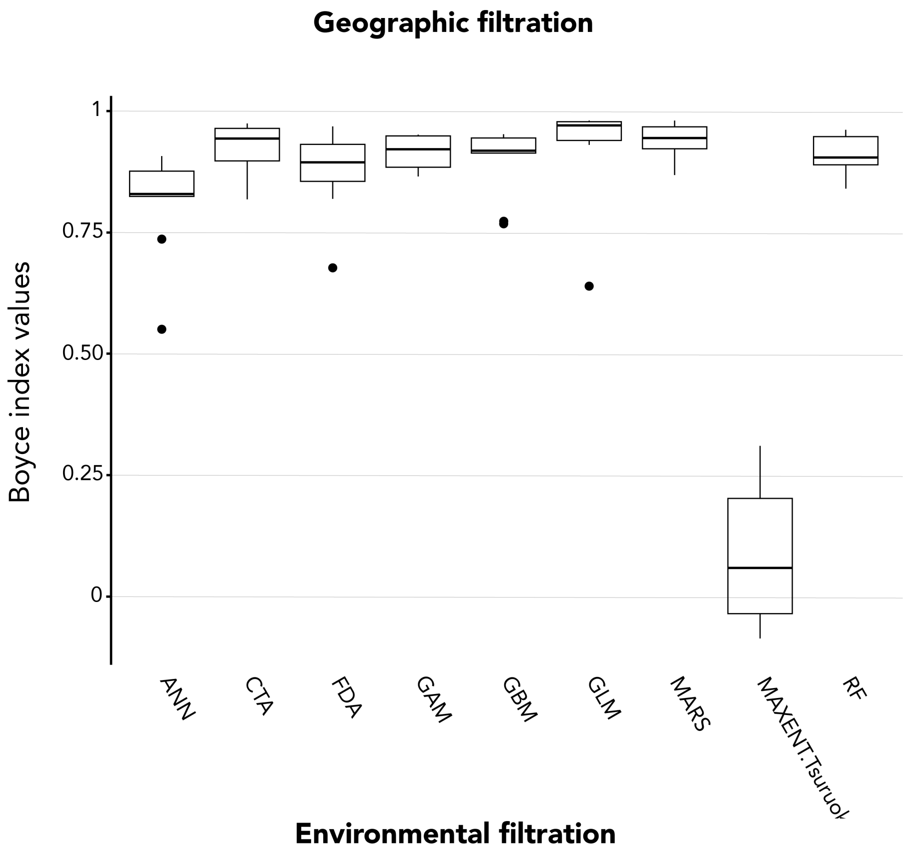


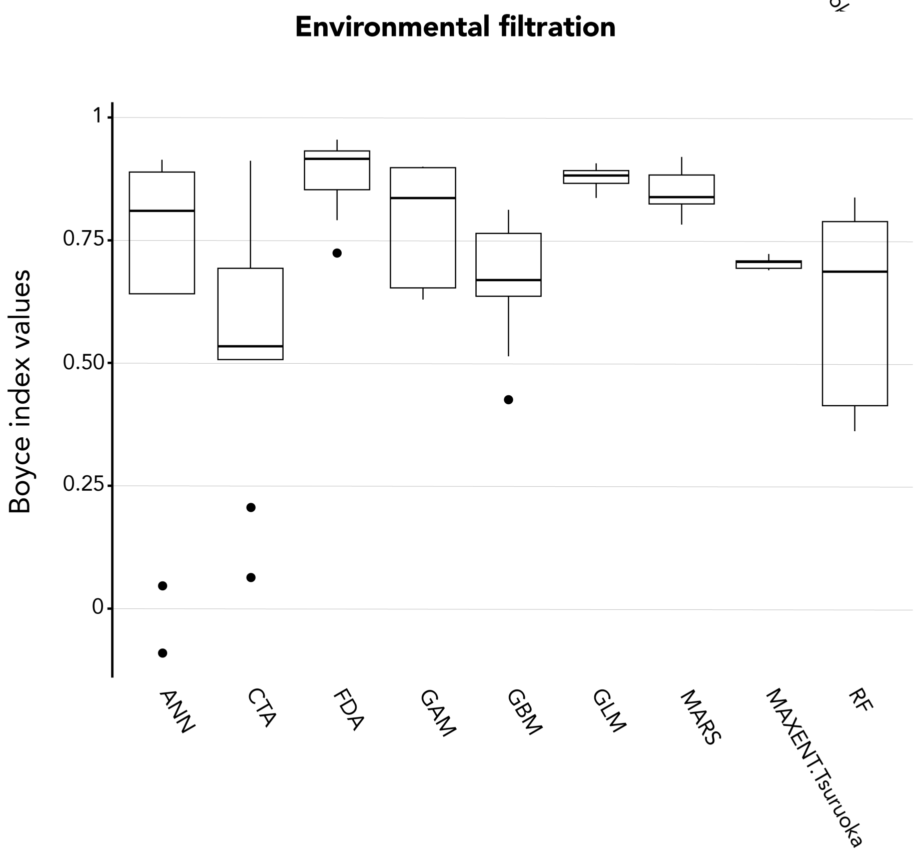

Method S1

Protocol of selection of variables significantly predicting the distribution of *Procyon lotor*.

We defined a protocol to identify variables that were not intercorrelated and significantly predicted the suitable range of environmental conditions for the species. This protocol is divided into three stages: first, we identified groups of intercorrelated variables on the basis of a hierarchical ascendant classification with a distance metric based on Pearson's correlation coefficient. Secondly, we identified the variables best predicting the distribution of the considered species for each group of intercorrelated variables. Thirdly, out of the variables saved at stage 2 and the variables not correlated to any other, we kept the variables that best predicted the distribution of *Procyon lotor*.

Because our modelling protocol is based on different modelling techniques with various criteria, the predictive power of each variable is difficult to assess and compare between models. Therefore, we applied a method that allows the importance of each variable to be assessed independently of the modelling technique. This method is implemented in BIOMOD as the « variable importance » procedure. The procedure for assessing the importance of a variable for a model is as follows: first, the model is calibrated with all the variables. Secondly, a standard prediction is made on the basis of all the variables. Thirdly, the assessed variable is randomized, and a new prediction is made with the randomized variable. Finally, the correlation between the standard prediction and the prediction with the randomized variable is calculated. If the correlation is high, then the considered variable contributes poorly to the prediction; then the predictive power of the variable is low. Conversely, if the correlation is low, then the predictive power of the variable is high. For each variable, this randomization procedure is computed 10 times. Hence, this procedure provides the average predictive importance of each variable for each of the computed models.

Step 1. Identification of groups of intercorrelated variables

We first calculated the Pearson's correlation coefficients between the 19 bioclimatic variables (table 1) across the entire world. Then, we calculated the following distance metric among variables: d = (1-r) where r was Pearson's correlation coefficient. On the basis of these distances, we constructed a hierarchical ascendant classification within which we identified groups of variables that were intercorrelated at a threshold of 0.7 (i.e. distance<0.3). Seven groups of intercorrelated variables were identified: (1) bio15, (2) bio2; (3) bio8, bio5 and bio10; (4) bio9, bio1, bio6, bio11, bio3, bio4 and bio7; (5) bio18, bio12, bio13, and bio16; (6) bio19; (7) bio14 and bio17.

Step 2. Reduction of variables in groups of intercorrelated variables

For each group of intercorrelated variables, we calibrated all the models of our modelling protocol. We made 3 pseudo-absence runs with 682 pseudo-absences selected for each run (number equal to the presences). For each run, the variable importance procedure was computed, and we selected the variable with the highest rank among all the models.

Step 3. Final selection of variables

Finally, we calibrated all the models with the variables selected at step 2 and the variables that were not correlated to any other, using the same protocol as in step 2. We then selected the variables presenting importance strictly superior to 0.10 for at least 50% of the models.
